# Supplementary material for: DMRforPairs: identifying Differentially Methylated Regions between unique samples using array based methylation profiles
Source: BMC Bioinformatics. 2014 May 15;15:141. doi: 10.1186/1471-2105-15-141 (PMC4046028; doi:10.1186/1471-2105-15-141)
Supplement: Additional file 2 — DMRforPairs output for the comparison of A431-MCF7 and NA17018-NA17105. Please start from the HTML files in each folder. Available via the BMC Bioinformatics website. [file 1471-2105-15-141-S2.zip › 1394847754114233_MOESM2_ESM/A431_MCF7/figures/20206.pdf]

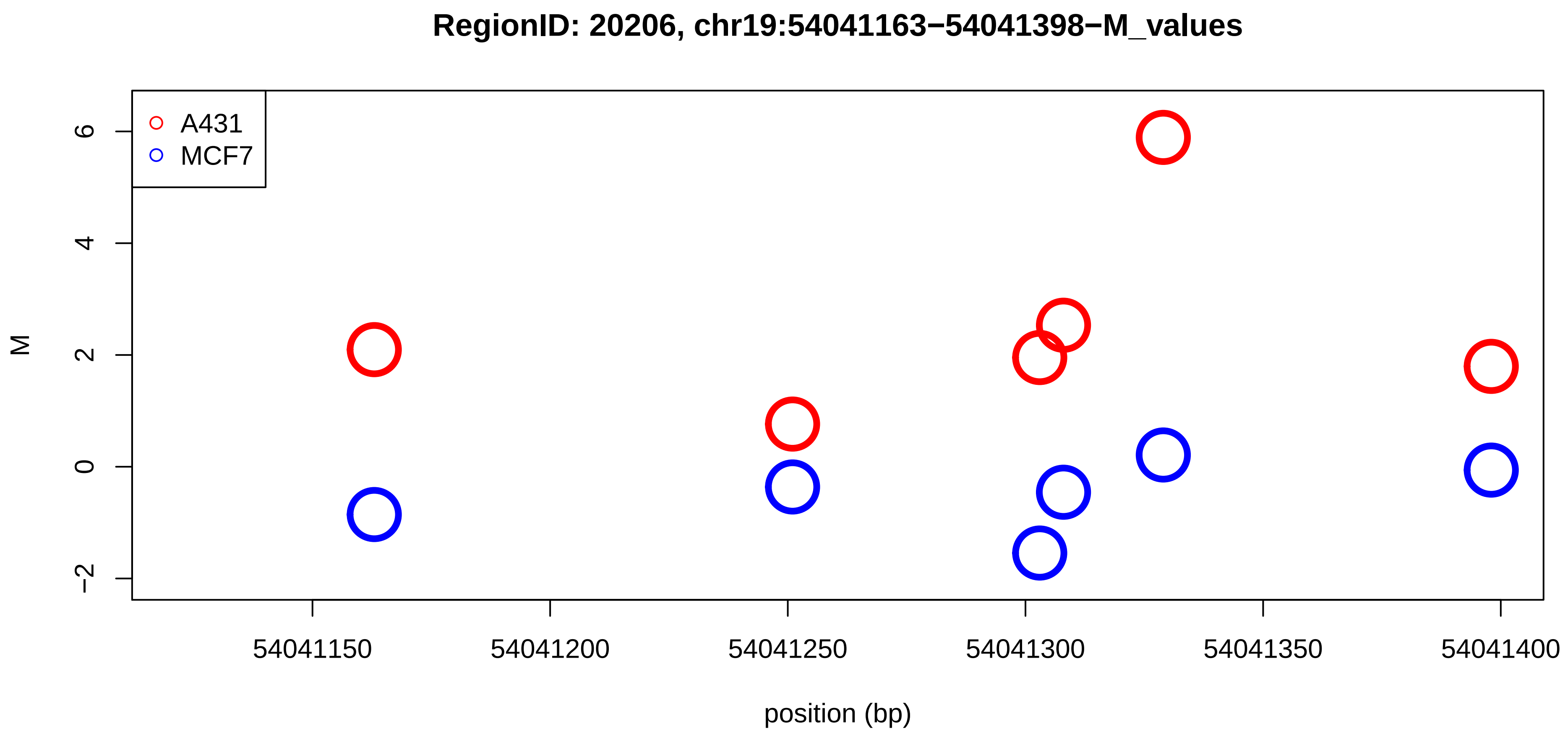
RegionID: 20206, chr19:54041163−54041398−M_values
A431
MCF7
M
6
4
2
0
−2
54041150
54041200
54041250
54041300
54041350
54041400
position (bp)

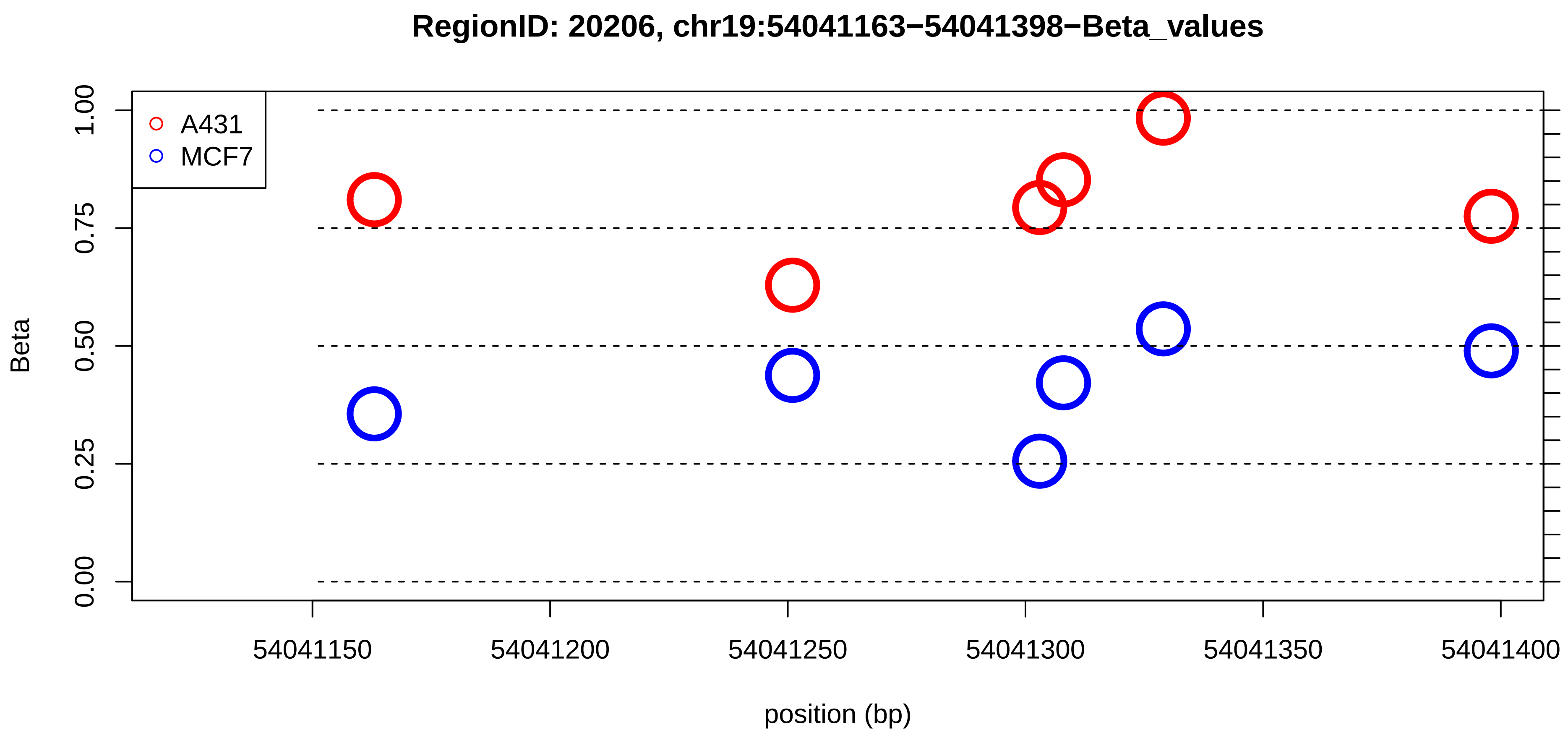
RegionID: 20206, chr19:54041163−54041398−Beta_values
A431
MCF7
Beta
1.00
0.75
0.50
0.25
0.00
54041150
54041200
54041250
54041300
54041350
54041400
position (bp)

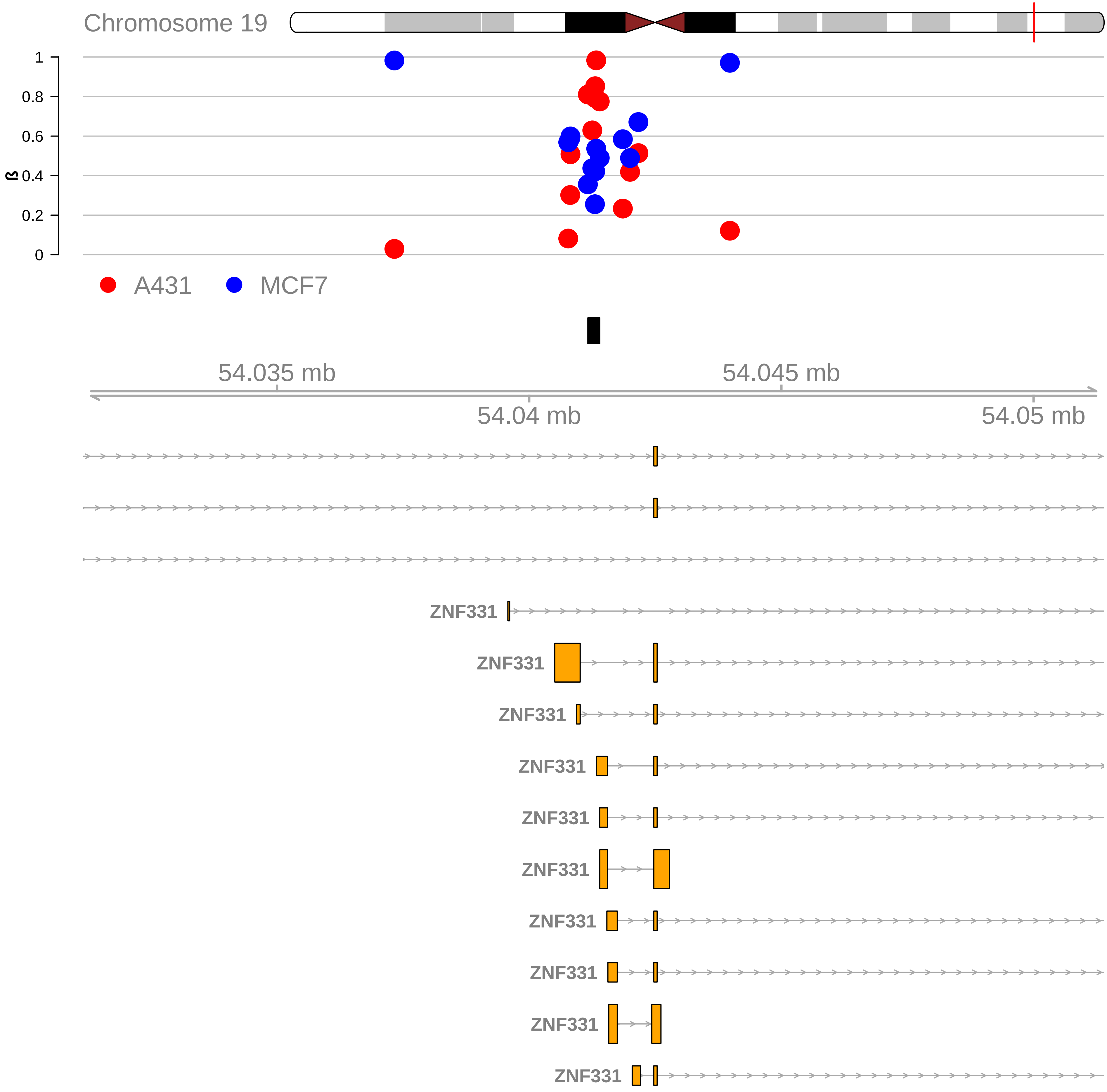

Chromosome 19
1
0.8
0.6
ß 0.4
0.2
0
A431
MCF7
54.035 mb
54.045 mb
54.04 mb
54.05 mb
ZNF331
ZNF331
ZNF331
ZNF331
ZNF331
ZNF331
ZNF331
ZNF331
ZNF331
ZNF331
